# High connectivity and genetic diversity in the Endangered endemic white-spotted sand bass ‘camotillo’ (*Paralabrax albomaculatus*) within the Galápagos Marine Reserve

**DOI:** 10.1186/s12862-026-02512-0

**Published:** 2026-06-18

**Authors:** Sarah M. Griffiths, Alicia C. Bertolotti, Pelayo Salinas-de-León, Rebecca Morris, N. Kobun Truelove, Stephen Box, Richard F. Preziosi

**Affiliations:** 1 Department of Natural Sciences, Manchester Metropolitan University, Manchester, UK; 2 Faculty of Life Sciences, University of Manchester, Manchester, UK; 3 Charles Darwin Research Station, Santa Cruz, Galápagos Islands, Ecuador; 4 Monterey Bay Aquarium Research Institute, Moss Landing, CA USA; 5 Blue Ventures, Bristol, UK; 6 School of Biological and Marine Sciences, University of Plymouth, Plymouth, UK

**Keywords:** Population genetics, Small-scale fishery, Marine protected area, Serranidae

## Abstract

**Background:**

*Paralabrax albomaculatus*, known as the camotillo or white-spotted sand bass, is a finfish endemic to the Galápagos Islands. An important species in the artisanal fishery, *P. albomaculatus* has undergone heavy population declines in recent decades, and is categorised as Endangered by the IUCN. Despite its socio-economic importance and endemic status, nothing is currently known about the population structure of the species, impeding evidence-based fisheries management. In this study, we use microsatellite markers to investigate its population structure and genetic diversity over the east, south and west of the Galápagos archipelago.

**Results:**

We found evidence of high connectivity across the archipelago, with the species constituting a single population. We also found that genetic diversity was high, despite fishing pressure and recorded ongoing population declines.

**Conclusions:**

Our results suggest that *P. albomaculatus* should be managed as a single fishery stock across the Galápagos. As a single-population species and fishery, managers should also be aware that *P. albomaculatus* is inherently vulnerable to perturbation as recruitment from other populations is not possible in the event of population decline. However, high genetic diversity gives cause for cautious optimism regarding the genetic capacity for resilience of the population to novel environmental stressors. These results should aid in the implementation of a species-specific management plan for this Galápagos endemic.

**Supplementary Information:**

The online version contains supplementary material available at https://doi.org/10.1186/s12862-026-02512-0.

## Background

Fisheries provide an essential source of nutrition and income for human populations across the globe [1, 2]. However, widespread overexploitation of fishery resources, along with loss and degradation of habitat, pollution, harmful algal blooms, climate change, and invasive species (amongst other pressures), have resulted in population declines in many fished species [3–8]. This has serious impacts on human wellbeing through the loss food security and livelihoods [9, 10], as well as the loss of ecosystem function, resilience, and biodiversity [11–13].

Effective fisheries management is essential in enabling sustainable resource extraction, avoiding overexploitation, and promoting stock recovery where overfishing has taken place. To do so, understanding the spatiotemporal boundaries of discreet fishery stocks is crucial for defining the scale of management required and implementing coherent strategies across a population’s range [14]. However, this is not intuitive for highly mobile marine species or those with a pelagic life stage, due to the seemingly physically ‘open’ nature of the environment. In addition, many marine species have a planktonic larval phase where they can disperse for up to several months in ocean currents before settlement, which can range from being close to spawning locations to hundreds of kilometres away [15]. As larvae are almost impossible to track in the field due to their small size [16], it is difficult to identify source-sink population dynamics and thus discrete fishery stocks. Furthermore, dispersal interacts with ocean circulation patterns and environmental conditions to shape population structure patterns, adding further complexity to attempts to predict population structure. Biophysical models - ocean current models in combination with biological data - can reveal physical connectivity patterns and predict larval source-sink dynamics, and as such is an effective tool for spatial management planning [17, 18]. However, empirical data remains important, especially where limited life-history or oceanography data are available.

Neutral genetic markers can be used to identify distinct population units and identify the degree of dispersal between disparate geographic locations [19, 20]. When locations are connected by dispersal, gene flow results in increasing genetic homogenisation; conversely, isolated locations that experience little immigration or emigration undergo genetic drift over time, becoming more genetically distinct. Population genetic information can therefore be used in fisheries management to identify stocks, important sources of larvae that recruit to disparate geographic locations, or areas with potential to act as pathways or stepping stones between populations [21]. This information can be incorporated into management, such as stock assessments and marine protected area (MPA) planning, to improve fishery sustainability [22, 23].

Genetic diversity itself is also an important parameter for population health and resilience. Overfished populations can exhibit losses in genetic diversity [24, 25] (although this is not consistent across taxa; [26]), making them vulnerable to future stressors such as disease and temperature changes due to a loss of adaptive capacity [27]. Isolated populations are most vulnerable to genetic diversity losses, as they lack connectivity with other populations and thus opportunities for new genetic variation to enter the population through immigration to counter the effects of genetic drift. Consequently, fragmentation of populations caused by fishing pressure could also impede recovery or maintenance of genetic diversity, as well as population numbers, through losses of connectivity pathways [28].

This study focuses on the camotillo, or white-spotted sand bass, *Paralabrax albomaculatus* (Jenyns, 1840), a bony fish belonging to the subfamily Serraninae (family Serranidae). *Paralabrax albomaculatus* is endemic to the Galápagos Islands, and is an important species for the local artisanal fishing industry, supporting around 1,200 artisanal fishers [29–31]. However, fishing pressure has resulted in severe population declines, with an estimated reduction in population size of between 50 and 80% since the 1990s, based on historical catch data [32]. As a result, and due to continued fishery pressure, the species is listed as ‘Endangered’ on the IUCN Red List [32]. It is therefore imperative that fishery managers have access to the necessary data to enable sustainable and effective management of the fishery. However, there is currently no information available regarding the species’ population structure and genetic status across the archipelago.

This study uses microsatellite markers to investigate population genetics of *P. albomaculatus* across the Galápagos Marine Reserve (GMR) with the aim of providing information to aid fishery and marine protected area management. Particularly, we aim to (1) characterise the population genetic structure of the species within the archipelago; (2) identify any barriers to gene flow and dispersal; and (3) quantify genetic diversity in the population.

## Methods

### Study site

The Galápagos Islands are located approximately 1,000 km from the coast of Ecuador, South America. Galápagos marine ecosystems and resources are managed as part of the Galápagos Marine Reserve (GMR), which is one of the largest marine protected areas in the world, covering 133,000 km^2^. Habitat within the GMR is primarily composed of rocky reefs at varied depths, while the oceanographic environment is dominated by the major currents – the warm Panama current running from the north (affecting mainly the northern islands), the cold Humboldt current running from the south (affecting mainly the southern and central islands), and the cold eastward Equatorial Undercurrent (or Cromwell current), an upwelling of cold productive waters in the west of the archipelago [33]. This confluence of currents creates a highly heterogeneous environment in water temperature and nutrient concentrations, creating a diverse array of niches and biotic communities across the archipelago [34–36].

### Study species

**Fig. 1:**
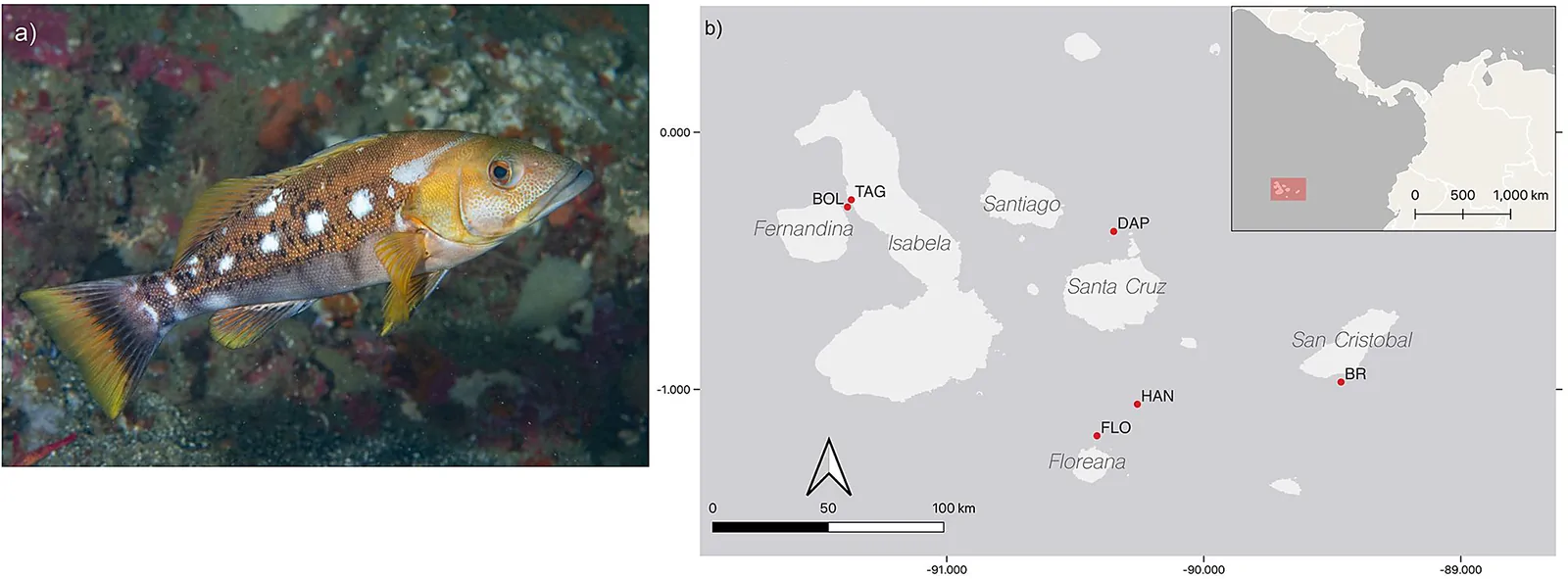
**a**) Photograph of *Paralabrax albomaculatus* © Frank Krasovec https://creativecommons.org/licenses/by-nc/4.0/. **b**) *Paralabrax albomaculatus* sampling locations used in this study. Red circles indicate where samples were collected: BOL: Canal Bolivar; TAG: Tagus Cove; BR: Banco Ruso; DAP: Daphne; FLO: Floreana; HAN: Hancock Bank; Map created in QGIS v3.20 using ESRI and Natural Earth basemaps

*Paralabrax albomaculatus* (Fig. 1a) occurs in rocky reef, sandy and seamount habitats through the Galápagos from the shallows to over 200 m in depth [31]. It exhibits a preference for cooler waters, and is therefore found in deeper reefs and seamounts over 50 m in depth across the archipelago, apart from off the western coasts of the islands Fernandina, Isabela and Floreana, which are influenced by the cold Equatorial Undercurrent, where the species is common on shallow reefs.

The camotillo is functionally gonochoristic and reproduces sexually. It spawns between October and March, with a peak of reproductive output between November and January [37]. This coincides with the transition from the cold to warm season in the Galápagos, where the current regime shifts from domination of the Humboldt and Cromwell currents to the Panama current. It is unknown whether the camotillo forms spawning aggregations, and the pelagic larval stage is of unknown duration.

### Sample collection

A total of 131 samples of *P. albomaculatus* were collected from six locations across central, southern and western parts of GMR in 2011–2012 (Fig. 1b; Table 1). Tissue samples (liver or muscle) were taken post-mortem from specimens captured by artisanal fishermen, and preserved in 90% ethanol and stored at -20 °C until DNA extraction. All specimens were captured for use in the artisanal fishery; sampling was opportunistic.

**Table 1:** Sampling details, genetic diversity statistics and average inbreeding coefficients in *Paralabrax albomaculatus*

Sampling location	Sample site name	Site code	*n*	Longitude	Latitude	*H*_O_	*H*_E_	*F*_IS_	AR (± SEM)
Canal Bolivar	Canal Bolivar	BOL	30	-91.38633	-0.2904319	0.925	0.909	-0.017	8.56 ± 0.88
Isabela/ Canal Bolivar	Tagus Cove	TAG	9	-91.37157	-0.262544	0.910	0.919	0.011	9.34 ± 0.90
San Cristobal	Banco Ruso	BR	40	-89.465958	-0.971676	0.834	0.903	0.076	8.87 ± 0.81
The Daphnes	Daphne	DAP	14	-90.350256	-0.385856	0.895	0.907	0.013	9.14 ± 1.00
Floreana	Floreana	FLO	14	-90.41553	-1.180703	0.873	0.919	0.050	9.2 ± 0.90
Hancock bank	Hancock	HAN	24	-90.258382	-1.057813	0.819	0.910	0.100	9.16 ± 1.04

Genetic diversity statistics (observed heterozygosity (*H*_O_), expected heterozygosity (*H*_E_), inbreeding coefficient (*F*_IS_) and allelic richness (*AR*)) are averaged over seven microsatellite loci. *n*: number of samples analysed; *H*_O_: Average observed heterozygosity; *H*_E_: Average expected heterozygosity; *F*_IS_: Average inbreeding coefficient; *AR*(± SEM): Average allelic richness (± standard error of the mean)

### DNA extraction and microsatellite genotyping

DNA was extracted from liver or muscle tissue samples using the DNeasy Blood and Tissue Kit (Qiagen) according to the manufacturer’s protocol. Ten microsatellites previously developed by (Bertolotti et al. [38] (Paxalb_10, Paxalb_4, Paxalb_32, Paxalb_22, Paxalb_11, Paxalb_1, Paxalb_20, Paxalb_24, Paxalb_8, Paxalb_35) were used to genotype the samples. Briefly, forward primers were labelled with either 6-FAM or VIC fluorophores and combined in the PCR duplexes described in Bertolotti et al. [38], and amplified using the Type-it Microsatellite PCR kit (Qiagen) according to the manufacturer’s protocol. PCR products were sized using capillary electrophoresis at the Smithsonian Institute, Washington D.C., USA, on a DNA Analyzer 3730 (Thermo Fisher Scientific) using a homemade ROX-based size standard. GeneMapper v.3.7 (Thermo Fisher Scientific) was used to manually score allele peaks. The package MsatAllele v.1.05 [39] in RStudio [40] was used to visualise the distribution of raw allele size values and aid manual binning into discrete allele size bins. Where alleles occurred outside of the motif repeat pattern (e.g. less than 4 base pairs apart for a tetranucleotide locus), or where bins only contained a small number of individual samples, electropherograms for those samples were cross-referenced and allele scoring re-checked to avoid erroneous scoring and binning. All analyses in RStudio were carried out using version 1.1.442, with R version 4.0.2 [41].

### Summary statistics and genetic diversity

The frequency of null alleles at each locus was calculated using the Expectation Maximisation Algorithm [42] as implemented in FreeNA [43]. Linkage disequilibrium between loci was tested using Genepop on the Web v.4.7.5 [44, 45] using default settings, and p values were corrected for multiple tests using the Benjamini-Yekutieli false discovery rate correction method [46] using the p.adjust function in R. Inbreeding coefficients (*F*_IS_) and probability of deviation from Hardy-Weinberg equilibrium (HWE) per locus per site were calculated in GenoDive v.3.01 [47], using the least-squares methods and 50,000 permutations to calculate p values. P values were corrected for multiple tests (per locus) using the Benjamini-Yekutieli correction as previously. Observed heterozygosity (*H*_O_) and expected heterozygosity (*H*_E_) were estimated per locus and per population in GenoDive v.3.0.1. Rarefied allelic richness (*AR*) per population was calculated with the ‘hierfstat’ package in R [48], and standard error of the mean was calculated within R.

### Population structure

Due to the relatively small number of loci utilised, limited sample sizes, and loci hypervariability, a power analysis was conducted using POWSIM v4.1 [49] to investigate the statistical probability that fine-scale population structure could be detected in this study. The model was run to simulate divergence levels of *F*_ST_ at 0.025, 0.02, 0.015, 0.01, 0.005, 0.0025 and 0.001. Simulations for each level of divergence were carried out using 1,000 replicates and an estimated *N*_*e*_ of 1,500, using our sample sizes and observed allele frequencies.

To examine the degree of genetic differentiation among the sites, pairwise *F*_ST_ and *D* [50] were calculated between sites using GenoDive, with significance tested using 50,000 permutations. The Bayesian model approach implemented in Structure v.2.3.4 [51] was used to investigate the presence of genetic clusters (*K*) in the dataset and proportionally assign each individual ancestry to those populations. The admixture and correlated allele frequency model was run with 200,000 burn-in repetitions followed by 1,000,000 Monte Carlo Markov Chain repetitions, using sampling locations as priors. Pritchard’s (2000) ln *Pr*(*X*|*K*) method was then used to determine the most likely value of *K*.

Pairwise Euclidean genetic distances between all individuals were calculated using the ‘dist’ function within RStudio. The resulting matrix was then used to conduct a Principle Coordinates Analysis (PCoA) in GenAlEx v.6.503 [52] to visualise genetic distance among individuals and identify the presence of any genetic clusters.

To test for correlation between genetic and in-water distances among sites (i.e. genetic isolation by distance), a Mantel test was performed using the *F*_ST_ matrix generated previously, and a matrix of least-cost in-water distances between sites created using the ‘marmap’ package [53] in R. The Mantel test was conducted using the ‘ade4’ package [54] in R, with 999 permutations to test significance.

An Analysis of Molecular Variance (AMOVA) was conducted in Genodive v.3.01 to partition genetic variance in the dataset within individuals, within sites, and among sites (no wider grouping hypotheses were tested). The AMOVA was run using the infinite allele model, with 1,000 permutations to test significance.

## Results

### Summary statistics and genetic diversity

All ten loci were successfully amplified. However, two of the loci (Paxalb_32 and Paxalb_8) were excluded from further analysis due to significant linkage disequilibrium with other loci (*p* < 0.05 after Benjamini-Yekutieli correction), leaving eight loci in linkage equilibrium. Number of alleles per locus ranged from 9 to 71 (median 25.5), and observed (*H*_O_) and expected heterozygosity (*H*_E_) per locus were high, ranging 0.631 to 0.945 (median 0.884) and 0.779 to 0.984 (median 0.924) respectively (Table S1). Average null allele frequency was ≤ 3.1% in all loci except Paxalb_24, which was 11.2% (Table S1). Paxalb_24 also showed significant deviation from HWE in five of the six sites (*p* < 0.05 after Benjamini-Yekutieli correction) (Table S2). Paxalb_24 was therefore excluded from further analyses, yielding a dataset of seven loci. No other loci significantly deviated from HWE at any of the sites (Table S2), and average *F*_IS_ ranged from − 0.017 in Canal Bolivar (BOL) to 0.100 in Hancock Bank (HAN) (Table 1).

Observed heterozygosity (*H*_O_) per site (averaged over all loci) ranged from 0.819 to 0.925, while average expected heterozygosity (*H*_E_) ranged from 0.903 to 0.919 (Table 1). Rarefied allelic richness was consistent among sites, ranging from 8.56 ± 0.88 in Canal Bolivar (BOL) to 9.34 ± 0.90 in Tagus Cove (TAG) (Table 1).

### Population structure

The power analysis carried out using POWSIM software suggested that population differentiation of up to *F*_ST_=0.005 could be detected with a probability of 0.97, while an *F*_ST_ of 0.01 or above could be detected with a probability of 1; *p* < 0.05) (Figure S1). *F*_ST_ values were non-significant (*p* > 0.05) among all pairs of sites (Table 2), ranging from − 0.005 between Hancock (HAN) and Canal Bolivar (BOL), to 0.016 between Daphne (DAP) and Tagus Cove (TAG). Pairwise Jost’s *D* were also non-significant among all sites (Table 2), with values of 0.157 and 0.124 between Tagus Cove (TAG) and Daphne (DAP), and Tagus Cove (TAG) and Floreana (FLO) respectively, while all other pairwise comparisons were low, ranging − 0.032 to 0.059.

**Table 2:** Pairwise genetic differentiation among sites in *Paralabrax albomaculatus*

	BOL	BR	DAP	FLO	HAN	TAG
BOL	-	-0.015	0.059	0.007	-0.051	0.000
BR	-0.001	-	0.042	-0.009	-0.032	0.055
DAP	0.006	0.004	-	0.028	0.042	0.157
FLO	0.001	-0.001	0.003	-	0.006	0.124
HAN	-0.005	-0.003	0.004	0.000	-	-0.009
TAG	0.001	0.006	0.016	0.013	-0.001	-

*F*_ST_ is shown below the diagonal, and Jost’s *D* is shown below the diagonal. None of the values were statistically significant (*p* > 0.05). BOL: Bolivar; TAG: Tagus Cove; BR: Banco Ruso; DAP: Daphne; FLO: Floreana; and HAN: Hancock Bank

**Fig. 2:**
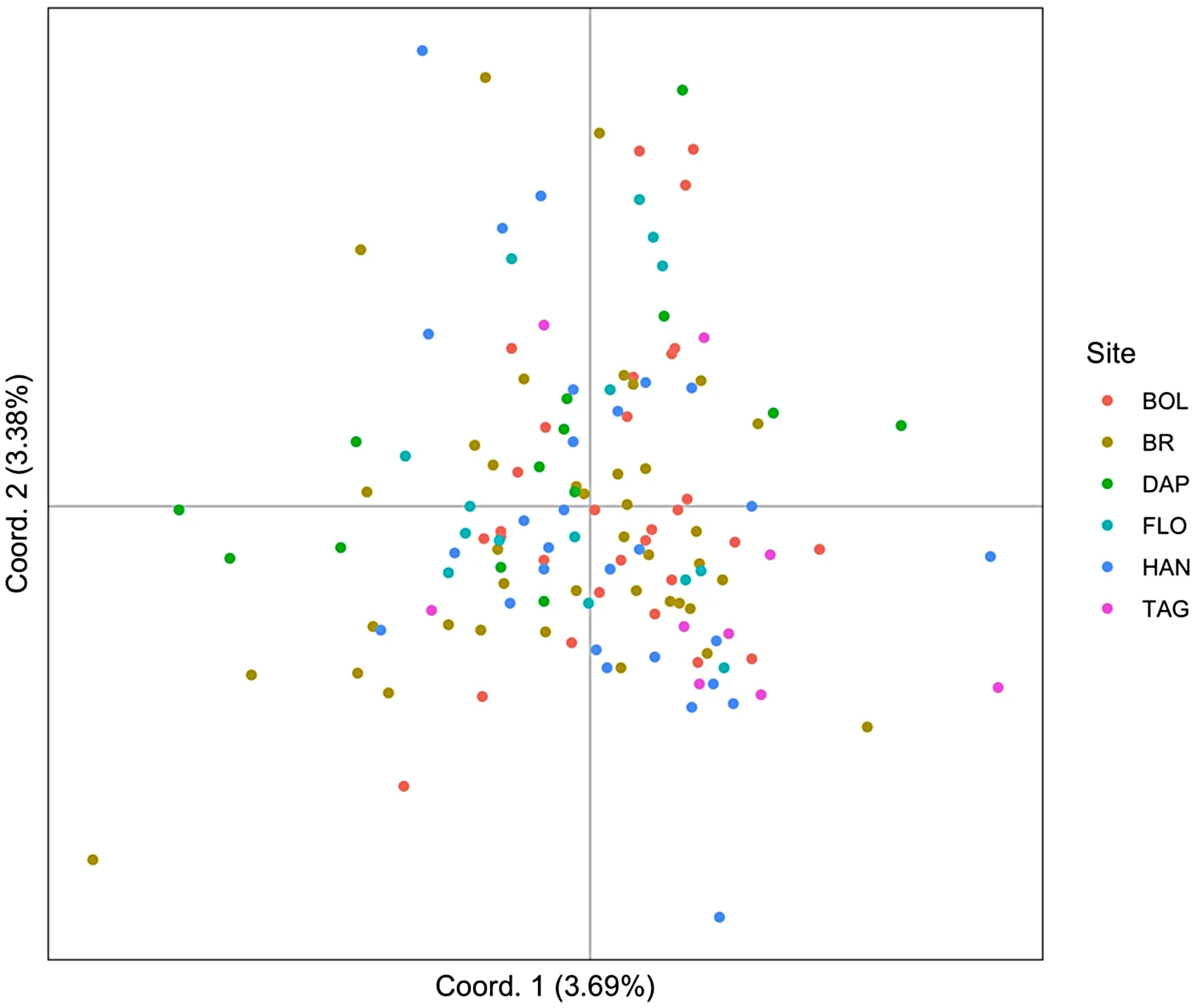
Principal Coordinates Analysis (PCoA) created using Euclidean distances between multilocus genotypes of *Paralabrax albomaculatus* individuals. Each point represents an individual; colour indicates sampling location according to inset legend. BOL: Canal Bolivar; TAG: Tagus Cove; BR: Banco Ruso; DAP: Daphne; FLO: Floreana; HAN: Hancock Bank

Analysis of ln *Pr*(*X*|*K*) values from the Structure run suggested *K* = 1 as the most likely scenario for the data, indicating only one genetic cluster is present (Figure S1). The PCoA (Fig. 2) was consistent with this result, showing individuals from all sites mixed together and no separation of any points into distinct clusters. The PCoA explained only 7.06% of the variance in the data in the first two axes.

**Fig. 3:**
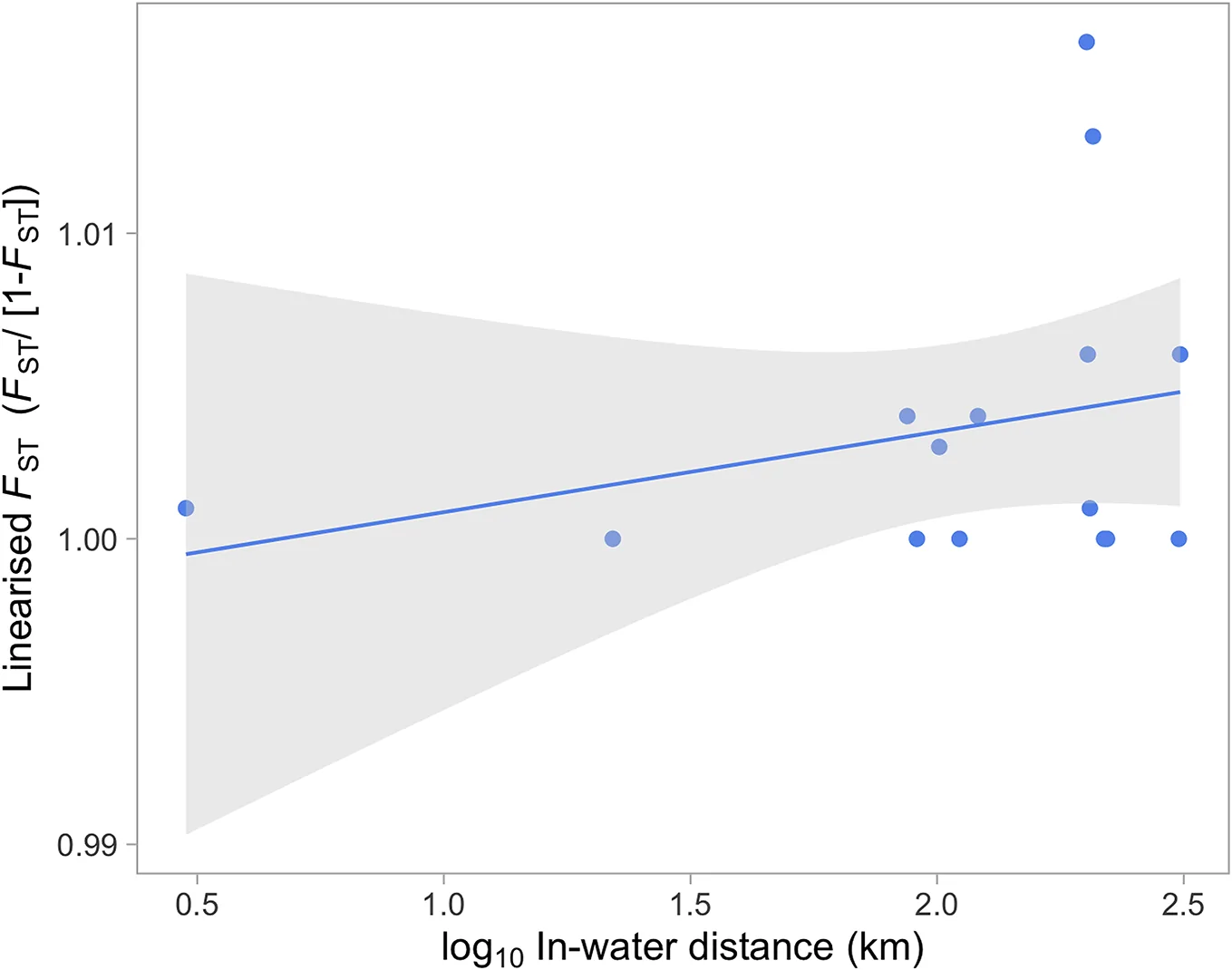
Genetic distance by in-water distance among *Paralabrax albomaculatus* sampling sites (*R* = 0.191, *p* = 0.221). Genetic distance is linearised *F*_ST_ (*F*_ST_/1-*F*_ST_) and in-water distance is log-transformed least-cost in-water distance

The Mantel test for isolation by distance was non-significant (*R* = 0.191, *p* = 0.221), showing no relationship between genetic and in-water distances between sites (Fig. 3).

The AMOVA (Table 3) showed that most of the genetic variation in the data was found within individuals (97.4%). 2.5% of genetic variation was among individuals within sites (*p* = 0.008), while only 0.2% was found between sites; this was found to be non-significant (*p* = 0.356).

**Table 3:** Analysis of Molecular Variance (AMOVA) showing partition of genetic variance in *Paralabrax albomaculatus* among individuals and sites in the Galápagos Islands

Source of variation	SSD	Variance components	% variance	*p* value
Within individuals	393.500	3.115	97.4%	-
Between individuals within sites	394.432	0.081	2.5%	**0.008**
Between sites	16.836	0.002	0.2%	0.356

Significant *p* values in bold

SSD: Sum of squared differences

## Discussion

### Population structure

This study shows strong evidence for high genetic connectivity across the Galápagos archipelago in *P. albomaculatus*. The analyses were consistent in showing that the locations sampled consist of a single panmictic population, indicating high gene flow and a lack of barriers to dispersal. *F*_ST_ and Jost’s *D* values indicated a slightly higher amount of differentiation between Tagus Cove and Daphne, and Tagus Cove and Floreana, than between any other pairs of populations. However, these were not statistically significant, and Structure and PCoA analyses did not indicate any differentiation of these sites. The slightly higher *F*_ST_ and *D* values are likely to be driven by the low sample size at Tagus Cove (*n* = 9) coupled with high allelic richness of the loci causing stochastic small differences in allele frequencies, rather than a true biological signal of population differentiation.

High connectivity in *P. albomaculatus* is likely to be driven in part by its pelagic larval phase; whilst its duration is unknown, species with a planktonic larval phase generally disperse more widely than direct developers or non-planktonic larvae, as larvae have the potential to be transported long distances in ocean currents [55]. Importantly, *P. albomaculatus* has a limited distribution; coupled with larval dispersal, high connectivity across its range is not surprising. Generally, longer pelagic larval durations (PLDs) are associated with wider dispersal [15] and more connected populations [56]. However, larval duration alone is often a poor predictor of dispersal capacity [15, 55], with larval behaviours contributing significantly to dispersal distances. Vertical distribution in the water column is significant in influencing larval transport due to differences in currents at different depths [15, 57]. Downward vertical migration to the benthos has been shown to limit dispersal to < 1 km even in species with longer pelagic larval durations of up to 30 days, whereas larvae that avoid the benthos have the potential to achieve longer dispersal distances (> 20 km) [15].

While the PLD in *P. albomaculatus* is unknown, congeners *P. nebulifer* and *P. maculatofasciatus* have mean PLDs of ~ 26 days and ~ 33 days respectively [58]. Similarly, larval swimming behaviours in *P. albomaculatus* have not been studied, but other species in the subfamily Serraninae have been noted to show age-related depth stratification [59], indicating vertical migratory behaviour. Particularly, Serraninae larvae have shown upward migration with age, which may enable them to disperse more widely [59]. Swimming speed and orientation behaviours can also impact dispersal distances [60, 61]; more research into larval biology and behaviours in *P. albomaculatus* will allow a more comprehensive understanding of the drivers of the high connectivity observed in this study.

Reproductive behaviours may also contribute to connectivity patterns [62], with large, concentrated spawning aggregations potentially allowing substantial genetic mixing and thus reduced population structure; for example, two Indo-Pacific grouper species with large spawning aggregations (*Epinephelus polyphekadion*, *Plectropomus areolatus*) were found to have panmictic populations, in contrast to the more structured populations of a grouper with a similar life history but multiple smaller spawning aggregations (*Plectropomus leopardus*) [63]. *Paralabrax albomaculatus* is known to have higher reproductive output in the warmer season between November and January, but it is unknown as to if they form large spawning aggregations, as reported in another regionally endemic and locally exploited species (*Mycteroperca olfax*) [64]. If so, this would be another potential mechanism for dispersing widely, resulting in high connectivity.

Movement during the adult life phase can also be a significant driver of population structure [65]. *Paralabrax albomaculatus* exhibits clear preferences for cooler waters in its distribution across the Galápagos, favouring the shallows only in the western parts of Isabela and Floreana, which are subject to the cold Cromwell upwelling subcurrent. Elsewhere, the species occupies deeper water, where it is cooler [31]. Habitat use or preference can create barriers to gene flow [66], however, *P. albomaculatus* appears to be able to overcome this, potentially due to wide dispersal at the larval stage. *Paralabrax albomaculatus* may also be able to move between locations (including to spawning aggregations) despite temperature gradients, perhaps by avoiding warmer waters through utilising different depths.

In addition to life history traits, oceanographic conditions are also important in driving patterns of population genetic structure. Ocean currents can connect subpopulations through creating dispersal pathways [67], while gene flow barriers can be produced by oceanographic currents [68], upwelling [69], bathymetry [70], physical obstacles [71], and abiotic parameters such as temperature and salinity [72]. The Galápagos marine environment is influenced by a complex system of currents (described in the Methods), and is characterised by spatial heterogeneity in abiotic characters such as temperature and nutrient concentration. Analysis of both biological assemblages [36] and physical abiotic parameters [34] have identified five distinct marine ecoregions across the archipelago (our sampling was conducted across two of the five). These could form barriers to dispersal in some species; indeed, spatial population structure associated with these ecoregions has been found in other marine species within the Galápagos [73, 74]. However, we found no evidence of population structure in *P. albomaculatus*, suggesting its life history traits and behaviours promote wide dispersal even despite complex ocean currents and considerable environmental heterogeneity.

While the evidence for a single panmictic population in *P. albomaculatus* was consistent among analyses, it is important to caveat that our study used only seven microsatellites, and many of these were highly polymorphic. Microsatellites typically have a lower power to detect fine-scale population structure compared to genome-wide single nucleotide polymorphism (SNP)-based techniques [75, 76], where vastly greater quantities of markers are used, allowing increased resolution. The number of microsatellites utilised in this study (seven) was lower than would ideally be used for population genetics; ten to twenty loci is generally considered to be more effective (though this depends on characteristics of the markers themselves, as well as the degree of structure exhibited by the study populations; in some study systems, lower numbers of markers have been found to give sufficient resolution) [77, 78]. Furthermore, while moderate levels of marker polymorphism can aid in population structure analysis by revealing finer-scale structure, hypervariability can mask fine-scale structure and bias allele frequencies due to the higher potential for size homoplasy, where higher mutation rates result in distinct alleles of the same size, which are erroneously scored as the same allele [79, 80]. It is possible, therefore, that subtle differences in population structure exist in *P. albomaculatus* which could not be discerned using the microsatellites utilised here. However, despite these potential issues, and the limited sample sizes available, our power analysis indicated that with the allele frequencies present in the population, the sample sizes and markers used in this study would be sufficient to detect population structure of up to *F*_ST_=0.005 with a probability of 0.97. This indicates that the set of markers used, though flawed, were nonetheless sufficient for population structure analysis and would be capable of detecting weaker levels of population structure if present. It is also important to note that sampling did not take place over the entire distribution of *P. albomaculatus*, with samples lacking from the northern islands of the archipelago and three of the five bioregions described by Edgar et al. [36]. Therefore, population structure may be present in areas of the species’ range which were not possible to sample in this study.

### Population structure of Galápagos fishes

Patterns of population structure in other Galápagos fishes have been found to be variable. High gene flow was also found using microsatellites in the misty grouper *Hyporthodus mystacinus* across the north west, central, and south east of the archipelago [81]. Similarly, no genetic structure was found using mitochondrial cytochrome *b* and control region haplotypes in the sailfin grouper (or balacao, *Mycteroperca olfax*), stalkeye scorpianfish (*Pontinus* sp.), and yellowfin tuna (*Thunnus albacares*) across western and southeastern locations [82]. Reef fish *Stegastes arcifrons*, *Stegastes beebei* and *Holacanthus passer* and rocky-shore dwelling *Malacoctenus zonogaster* also did not exhibit any evidence of population structure across Galápagos using mitochondrial control region sequences [83].

In contrast, the Galápagos shark (*Carcharhinus galapagensis*) was found to exhibit fine-scale population structure between east and southwestern Galápagos [73]. Similarly, two species of Galápagos-endemic blennies (*Dialommus fuscus*, *Lepidonectes corallicola*) - the Galápagos-endemic blue-banded goby (*Lythrypnus gilberti*) and the Panamic sergeant major (*Abudefduf troschelii*) - showed weak but significant pairwise *F*_ST_ values in mitochondrial control region sequences among different Galápagos localities [83]. Further work on the blue-banded goby using selectively-neutral SNPs confirmed these previous results [84]. In the Galápagos bullhead shark (*Heterodontus quoyi*), SNP analysis revealed fine-scale population structure across different depths, suggesting deeper water acts as a partial barrier to dispersal in this species [85]. The yellow snapper, *Lutjanus argentiventris* also exhibits signs of weak population structure, between the western and eastern, and northern and eastern, parts of the archipelago according to microsatellite analysis [86]. This variation in population structure patterns across fish species is likely to be the result of variation in behavioural and life history traits.

### Genetic diversity

We found high microsatellite genetic diversity in the *P. albomaculatus* population. Allelic richness, observed heterozygosity and expected heterozygosity were very high, averaging 9.0, 0.88, and 0.91 respectively over all sites and loci. This is high relative to other fish species; in a meta-analysis of fish genetic diversity, observed heterozygosity at microsatellite loci ranged from 0.46 to 0.84, averaging 0.64, across 22 species in the Serranidae family [26]. Observed microsatellite heterozygosity in another Galápagos serranid, the misty grouper (*Hyporthodus mystacinus*), was also considerably lower than in the present study, averaging 0.57 across the archipelago [81]. We also found no evidence of inbreeding, with no significant deviations from Hardy-Weinberg equilibrium in any sites or loci apart from Paxalb_24, which is likely to be due to the null alleles also present at the locus.

Given the population declines experienced by the species due to overfishing (estimated at 50%-80% in the last three decades; [32]), the high genetic diversity observed in this study may be considered surprising. Severe population declines generally result in genetic bottlenecks, where alleles are rapidly lost from the population [87], and genetic diversity has significantly declined in wild populations globally since the industrial revolution [88]. A meta-analysis by Pinsky and Palumbi [24] found that overfished populations had lower genetic diversity in 9 out of the 12 genera and families tested. However, the relationship between conservation status and genetic diversity is complex; a meta-analysis by Martinez et al. [26] found that genetic diversity levels were not significantly different between threatened and non-threatened fish species. This may be because genetic diversity reductions generally occur when populations experience severe population declines over very few generations, or in very small population sizes. Neither of these criteria necessarily apply to chronically overfished species, such as *P. albomaculatus*, or even in collapsed fishing stocks.

Other species in the Galápagos have shown mixed patterns of genetic diversity in relation to fishing status. The overfished regional endemics, the stalkeye scorpianfish (*Pontinus* sp.) and sailfin grouper (or bacalao, *M. olfax*), were found to have low haplotype and nucleotide diversity at the mitochondrial cytochrome *b* and D-loop genes [82]. Other finfish species (*S. rivoliana*, *Acanthosybium solandri*, *Thunnus albacares*) in the Galápagos did not show evidence of low genetic diversity in the same loci. However, these species are not Galápagos- nor regionally- endemic; therefore fishing pressure and management of these species throughout their population ranges will also impact their genetic diversity. As an endemic species that has experienced heavy population declines, it is remarkable that *P. albomaculatus* has apparently avoided a genetic bottleneck. This is even more so given its status as a single population, meaning genetic rescue via connectivity from other populations is not possible. 

As well as experiencing population declines due to overfishing, *P. albomaculatus* shows a skewed sex ratio, biased towards females in a 4.36:1 ratio [37]. Skewed sex ratios can cause lower than expected levels of genetic diversity due to the lower effective population size (N_*e*_), and can be a better predictor of genetic diversity than census population size [89, 90]. However, as a broadcast spawner, high fecundity in *P. albomaculatus* is likely to mitigate the effects of female-biased sex ratios on population size [91], and thus potentially buffering against negative impacts on genetic diversity.

Maintaining genetically diverse populations is recognised as an important component of resilience for marine species and their associated ecosystem services [92]. Genetic diversity is the foundation of adaptation, thus populations with higher genetic diversity are more likely to persist in the face of novel stressors. However, while high levels of genetic diversity in *P. albomaculatus* are cause for optimism, the loci utilised here exhibited unusually high levels of variability, with number of alleles per locus reaching 60 in Paxalb_1 and 71 in Paxalb_20, and median number of alleles per locus of 25.5). During initial development of the loci, screening was conducted based on polymorphism levels, with more polymorphic loci selected to allow for increased sensitivity to detecting population structure [38], a strategy commonly used in microsatellite development [93]. However, this can cause upwardly biased estimates of genetic diversity when microsatellite diversity is extrapolated to the genome-wide level [94]. Additionally, microsatellites are selectively neutral and therefore do not necessarily reflect genome-wide genetic diversity at functionally-important loci that could contribute to adaptation and persistence in the face of stressors [94, 95]. Further research into the genome of the species could identify functional genes relevant to predicted local stressors, such as thermal anomalies due to climate change, and disease [96]. This would enable a more accurate understanding of adaptability and plasticity of the species, and thus its future resilience. This would in turn allow a more accurate assessment of sustainability and resilience of the *P. albomaculatus* artisanal fishery.

## Conclusions

Our results suggest that *P. albomaculatus* should be managed as a single fishery stock across the Galápagos. Data on landings should therefore be compiled from across sites in the GMR when conducting stock assessments, and use of no-take zones or other restrictive measures should be coordinated spatially to take into consideration the whole population across the archipelago. It is also important for fishery managers and users to be aware that, as the entire species appears to constitute a single population, *P. albomaculatus* is unable to recover through larval replenishment or connectivity from other sources. Therefore, protection during reproduction, either through identification and protection of spawning aggregations or through closed seasons during the peak reproductive season, is key to ensuring population persistence over the whole archipelago.

Our study tentatively suggests that genetic diversity levels are robust, giving cause for cautious optimism over the genetic health of the species. However, as this is the first genetic assessment of the species, it is possible that genetic diversity levels were higher prior to modern population declines, and current levels represent a reduction from historical baselines. It is also possible that the high variability of the microsatellite loci does not reflect genome-wide diversity levels. Nonetheless, this study provides a current baseline against which the population may be monitored in the future. It is critical to maintain this genetic diversity to prevent loss of evolutionary potential in the species, and thus its ability to adapt to future stressors; the most effective way to maintain genetic diversity is to avoid population declines and fragmentation.

## Supplementary Information

Below is the link to the electronic supplementary material.

Supplementary Material 1

## Data Availability

The dataset supporting the conclusions of this article is available in the Manchester Metropolitan University eSpace research repository (https://doi.org/10.23634/MMU.00642014).
