# Supplementary material for: High connectivity and genetic diversity in the Endangered endemic white-spotted sand bass ‘camotillo’ (Paralabrax albomaculatus) within the Galápagos Marine Reserve
Source: BMC Ecol Evol. 2026 Jun 18;26:80. doi: 10.1186/s12862-026-02512-0 (PMC13573344; doi:10.1186/s12862-026-02512-0)
Supplement: Supplementary file 1 [file 12862_2026_2512_MOESM1_ESM.docx]

**Supplementary Information**

**Table S1: *Paralabrax albomaculatus* microsatellite loci characteristics: Total number of alleles per locus (NA) and their size range across the six sites tested in the study; average frequency of null alleles, observed heterozygosity (*H*_O_) and expected heterozygosity (*H*_E_) per locus over the six sites.** Microsatellite loci originally characterised in Bertolotti et al. (2015).

| Locus | *NA* | Size range (bp) | Null allele frequency | *H*_O_ | *H*_E_ |
| --- | --- | --- | --- | --- | --- |
| Paxalb_1 | 60 | 293-445 | 0.006 | 0.926 | 0.957 |
| Paxalb_4 | 26 | 410-506 | 0.016 | 0.874 | 0.917 |
| Paxalb_10 | 27 | 405-523 | 0.031 | 0.893 | 0.946 |
| Paxalb_11 | 22 | 177-259 | 0.011 | 0.838 | 0.864 |
| Paxalb_20 | 71 | 339-474 | 0.012 | 0.948 | 0.984 |
| Paxalb_22 | 25 | 367-437 | 0.010 | 0.902 | 0.930 |
| Paxalb_24* | 25 | 418-481 | 0.112 | 0.631 | 0.895 |
| Paxalb_35 | 9 | 393-414 | 0.017 | 0.750 | 0.779 |

bp: base pairs; * This locus was removed from subsequent analyses

**Table S2 – *F*_IS_ per site per locus in *Paralabrax albomaculatus*.** Bold values indicate a significant deviation from Hardy-Weinberg equilibrium (p<0.05 following Benjamini-Yekutieli correction for multiple tests). BOL: Bolivar; TAG: Tagus Cove; BR: Banco Ruso; DAP: Daphne; FLO: Floreana; and HAN: Hancock Bank.

|  | Paxalb_1 | Paxalb_4 | Paxalb_10 | Paxalb_11 | Paxalb_20 | Paxalb_22 | Paxalb_24* | Paxalb_35 |
| --- | --- | --- | --- | --- | --- | --- | --- | --- |
| BOL | 0.03 | 0.009 | -0.066 | -0.115 | 0.082 | -0.015 | **0.28** | -0.065 |
| BR | 0.024 | 0.030 | 0.076 | -0.088 | 0.017 | 0.03 | **0.396** | -0.024 |
| DAP | 0.03 | 0.125 | 0.171 | 0.029 | -0.035 | 0.141 | **0.316** | 0.075 |
| FLO | -0.053 | -0.005 | -0.061 | 0.158 | 0.129 | -0.067 | **0.307** | 0.006 |
| HAN | 0.079 | 0.075 | 0.031 | 0.179 | 0.025 | 0.02 | **0.226** | -0.067 |
| TAG | 0.084 | 0.052 | 0.188 | 0.027 | -0.007 | 0.072 | 0.244 | 0.354 |

* This locus was removed from subsequent analyses

Figure S1: Results from POWSIM [1] power analysis examining the probability (*P*(*A*)) of detecting population differentiation at various levels of population differentiation (*F*_ST_) with the seven microsatellite loci utilised in this study. *F*_ST_ values are the mean of 1000 replicate simulations, and the probability indicates the proportion of replicates in which the *F*_ST_ was significantly different from zero (p<0.05) in Fisher’s exact tests.

Figure S2: STRUCTURE [2] plots for *K*=1 (top) and *K*=2 (bottom) for *Paralabrax albomaculatus*. Each individual is represented by a vertical bar, and genetic clusters are represented by different colours. The proportion of each colour per bar reflects admixture proportions per individual. Plots were created using CLUMPAK[3]. K=1 had the highest statistical support based on Pritchard’s [1] ln(Pr(X|K) values. 1: Canal Bolivar, 2: Banco Ruso; 3: Daphne; 4: Floreana; 5: Hancock; 6: Tagus Cove.

**References:**

1. Ryman N, Palm S. POWSIM: A computer program for assessing statistical power when testing for genetic differentiation. Mol Ecol Notes. 2006;6:600–2. https://doi.org/10.1111/j.1471-8286.2006.01378.x.

2. Pritchard JK, Stephens M, Donnelly P. Inference of population structure using multilocus genotype data. Genetics. 2000;155:945–59. https://doi.org/10.1093/genetics/155.2.945.

3. Kopelman NM, Mayzel J, Jakobsson M, Rosenberg NA, Mayrose I. Clumpak: A program for identifying clustering modes and packaging population structure inferences across K. Mol Ecol Resour. 2015;15:1179–91. https://doi.org/10.1111/1755-0998.12387.
